# Ectopic cervical thymic cyst with thyroglossal duct cyst

**DOI:** 10.1016/j.bjorl.2024.101431

**Published:** 2024-04-02

**Authors:** Weiyao Chen, Juxing Sun, Yanqiu Zhou, Mengyin Li, Shaohua Wang, Xinxin Yang, Xiaoyu Li

**Affiliations:** a Clinical Medical College of Jining Medical University, Jining, P.R. China; b Affiliated Hospital of Jining Medical University, Department of Otolaryngology-Head and Neck Surgery, Jining, P.R. China; c Affiliated Hospital of Jining Medical University, Department of Anesthesiology, Jining, P.R. China

## Introduction

Cervical thymic cysts are uncommon in clinical practice, account for only 0.3% of all congenital neck cysts in children and only 1% of mediastinal tumors, and ectopic neck thymic cysts account for only 12.5% of thymic cysts.^1^ The most common congenital cervical cysts are thyroglossal duct cysts, so cervical thymus cysts are often misdiagnosed as thyroglossal duct cysts. The patient in our case had both a thymic cyst and a thyroglossal duct cyst, as well as the two disorders had distinct clinical symptoms and imaging.

## Case report

**Figure 1:**
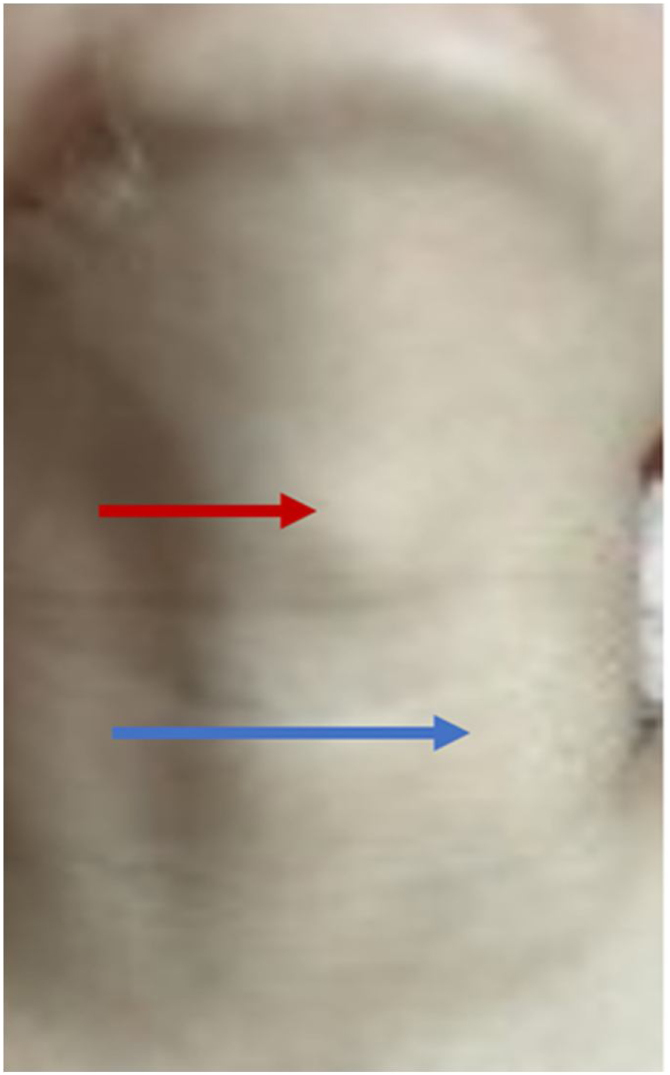
Mass between the thyroid cartilage and the cricoid cartilage (red arrow), body projection of anterior mass of the left carotid artery (blue arrow).

**Figure 2:**
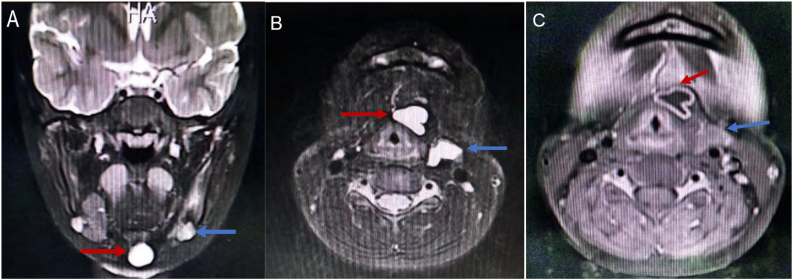
(A) Axial and (B) Coronal Magnetic Resonance Imaging (MRI) scan of the neck showed T2-weighted hyperintense signals seen between the hyoid (red arrow) and thyroid cartilage and in front of the left carotid artery (blue arrow). (C) Enhanced MR Imaging (DCE-MRI) scan of the neck showed the hyoid bone and the anterior thyroid cartilage lesions showed ring enhancement (red arrow) and the lesions in the left anterior carotid artery showed mild uneven enhancement (blue arrow).

A 7-year-old female was presented to the hospital with a neck mass that had been present for more than a year. On inspection, a 2 × 1 cm large mass was found in the middle of the neck below the left hyoid bone, with no redness or swelling on the skin's surface, no pain on palpation, and the mass was moved with swallowing (Fig. 1). Color Doppler ultrasound showed cystic echoes in the middle of the neck. Computed Tomography (CT) suggested a cystic lesion that was visible between the hyoid bone and thyroid cartilage, consistent with CT signs of a thyroglossal cyst, and an irregularly shaped cystic lesion with lobulated margins was seen anterior to the left carotid artery, adjacent to the common carotid artery. Magnetic Resonance Imaging (MRI) demonstrated cystic T1-weighted hypointense signals and T2-weighted hyperintense signals in the hyoid bone and thyroid cartilage and in front of the left carotid artery. After injection of contrast medium, the lesion in front of the hyoid bone and thyroid cartilage showed ring enhancement, and the lesion in front of the left carotid artery showed mild uneven enhancement (Fig. 2 A–C). The patient was previously fit, and her family had no history of genetic disease or similar conditions.

It was decided to excise both masses together after obtaining the consent of the parents of the child. The patient underwent a surgical procedure with general anesthesia in August 2022. Firstly, a transverse incision was made along the dermatoglyphic line on the surface of the thyroglossal cyst. Subsequently, the incision was extended to the left towards the surface of the left sternocleidomastoid muscle. The cervical vastus muscle was then incised and separated to expose the thyroglossal cyst. Upon observing that the root tip of the cyst was attached to the hyoid bone, complete excision of both the cyst and its connected portion of the hyoid bone measuring approximately 2 × 2 × 1 cm was performed. Subsequently, the second mass was excised, exposing the left sternocleidomastoid muscle initially and then carefully separating it from the ventral surface. The blood vessels and nerves were meticulously dissected to reveal a lobulated black cystic mass situated on the surface of the left carotid artery, pulsating synchronously with its arterial flow. With utmost caution, the mass was bluntly separated while ensuring the protection of the carotid artery to prevent any traction or injury. Ultimately, complete resection of the 3 × 2 × 1 cm-sized mass was achieved. Following meticulous saline irrigation of the operative site, negative pressure drainage was established for effective wound management before concluding with layered suturing.

**Figure 3:**
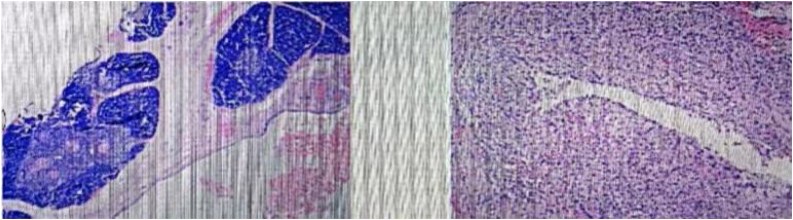
Microscopic examination shows that ectopic thymic tissue.

Postoperative pathologic revealed that the mass located between the thyroid cartilage and the cricoid cartilage was a thyroglossal cyst, whereas the mass located anterior to the left carotid artery was a thymic ectasia with cyst formation (Fig. 3). Then, the patient was prepared to be discharged from the hospital. The patient has been followed up for 13 months postoperatively and had no noticeable anatomic sequelae.

## Discussion

Ectopic thymus is a rare occurrence resulting from the descent of the thymus from the pharynx to the mediastinum, where any remaining tissue in a certain segment can lead to its formation.^2^ The anterior superior mediastinum is a preferred location for thymic cysts, while the base of the skull, neck, pharyngeal space, and pericardium are all favored sites for ectopic thymic cysts. The most common site of ectopic thymus is below the left thyroid gland.^3^ During the early stages of development, cervical ectopic thymic cysts do not exhibit any clinical manifestations. However, as these masses grow and enlarge due to the accumulation of cystic fluid, they exert pressure on surrounding blood vessels, nerves, trachea, esophagus, etc., leading to symptoms such as dyspnea, labored breathing, swallowing discomfort or dysphagia, hoarseness, choking sensation when drinking water, dizziness, fatigue, limb weakness, ptosis, and cervicofacial edema. Neck pain may also occur due to cyst growth. In our case, no clinical symptoms including pain were observed in this child.

Neck ultrasonography is performed, and the presence of a short line of strong echoes helps to detect an ectopic thymus. CT and MRI of the neck can also be performed, with clear borders, density similar to or slightly higher than that of the chest wall muscles, and homogeneous enhancement on its CT manifestation. MRI shows that it is higher than the fat on the T2 image, with homogeneous enhancement, and higher than the muscle on the T1 image, with low signal.^4^

It can be differentiated from gill slit cysts, lymphadenitis, lymphoma, lymphangioma, and other benign neck masses. It has been reported that ectopic thymic cysts in the neck can become cancerous.^5^ Pathologic diagnosis is the gold standard, and we ultimately rely on complete surgical excision of the mass to send the mass for pathologic examination to confirm the diagnosis.

## Conclusion

Ectopic thymic cysts of the neck, although rare, pose a diagnostic challenge for otolaryngologists. In this case report, we present an intriguing instance of a young girl with coexisting ectopic thymic and thyroglossal duct cysts, aiming to enhance understanding and management strategies for these distinct neck pathologies.

## Funding

This work was supported by the Shandong Provincial Natural Science Foundation (ZR2019MH059) and Shandong Provincial Medical and Health Science and Technology Development Plan (202307010435).

## Conflicts of interest

The authors declare no conflicts of interest.
